# Long-term continuous monitoring of arrhythmias in pigs with insertable cardiac monitors

**DOI:** 10.1007/s00424-024-02962-9

**Published:** 2024-05-04

**Authors:** Jonna Airaksinen, Satu Siimes, Juha Hartikainen, Seppo Ylä-Herttuala

**Affiliations:** 1 A.I.Virtanen Institute for Molecular Sciences, University of Eastern Finland, Kuopio, Finland; 2 Heart Center, Kuopio University Hospital, Kuopio, Finland

**Keywords:** Implantable cardiac monitor, Insertable cardiac monitor, Implantable loop recorder, Arrhythmia, Rhythm monitoring

## Abstract

Arrhythmia detection is essential when assessing the safety of novel drugs and therapies in preclinical studies. Many short-term arrhythmia monitoring methods exist, including non-invasive ECG and Holter. However, there are no reliable, long-term, non-invasive, or minimally invasive methods for cardiac arrhythmia follow-up in large animals that allows free movement with littermates. A long follow-up time is needed when estimating the impact of long-lasting drugs or therapies, such as gene therapy. We evaluated the feasibility and performance of insertable cardiac monitors (ICMs) in pigs for minimally invasive, long-term monitoring of cardiac arrhythmias that allows free movement and species-specific behavior. Multiple implantation sites were tested to assess signal quality. ICMs recognized reliably many different arrhythmias but failed to detect single extrasystoles. They also over-diagnosed T-waves, resulting in oversensing. Muscle activity and natural startles of the animals caused noise, leading to a heterogeneous signal requiring post-recording evaluation. In spite of these shortcomings, the ICMs showed to be very useful for minimally invasive long-term monitoring of cardiac rhythm in pigs.

## Background

Short-term methods are available for the monitoring of cardiac rhythm in small animals and in anesthetized pigs. However, there are no reliable, long-term, non-invasive, or minimally invasive methods for cardiac arrhythmia follow-up in large animals that allows free movement with littermates. Long follow-up time is needed when estimating the impacts of long-lasting drugs or therapies, such as gene therapy. Previously it has been shown, that insertable cardiac monitors (ICMs) can record tachyarrhythmias, such as ventricular tachycardias, leading to ventricular fibrillation in a porcine model [6]. However, we lack a thorough understanding of the suitability of ICMs to detect a wide variety of arrhythmias in a large animal model.

ICMs are small electronic devices that are used in humans to help arrhythmia diagnostics for example in unexplained syncope or palpitations. The device is implanted subcutaneously under local anesthesia and the procedure is only mini-invasive. ICMs record a single-lead surface ECG so they are not diagnostically as accurate as the golden standard 12 lead ECG, but in most cases, they are reliable enough for arrhythmia detection and diagnosis. Even though the device monitors cardiac rhythm constantly, it reacts only when a pre-programmed heart rate threshold is met and stores the arrhythmia episode in internal memory. In addition, patient-activated recordings and remote data transmission are possible, but not in animal studies. ICMs have a battery lifetime of 2–3 years, and therefore, they are suitable for a long-term follow-up of arrhythmias [3]. In this work, we tested the suitability of ICMs for long-term monitoring of arrhythmias in pigs.

## Material and methods

### Animals

Thirteen female pigs aged 3–4 months (39.5 ± 12.5 kg) were selected to the study. Female pigs were used because of their less aggressive behavior as compared to males. The animals were randomly divided into normoxic (*n* = 6) and ischemic (*n* = 7) groups, with different follow-up times. There was no reason to expect arrhythmias in the young, healthy, normoxic pigs and a follow-up time of 8 days was used for this group. However, ischemia might cause several types of arrhythmias and therefore we chose to extend the follow-up time to 22 days for the ischemic group. All animal procedures were approved by The National Animal Experimental Board of Finland and carried out in accordance with the guidelines of the Finnish Act on Animal Experimentation. Animals were kept in standard housing conditions in the National Laboratory Animal Center of the University of Eastern Finland, Kuopio, Finland.

The ischemic pig model was made by placing a bottleneck-shaped plastic tube in the LAD after the first diagonal. When the antithrombotic medication is stopped, the blood flow diminishes, creating ischemia to the area it once supplied blood to [8]. Angiography was performed twice for every animal. The first imaging was done at the beginning of the study alongside with the implantation of bottleneck stents and as a control procedure for the normoxic animals. Angiography was repeated at the end of the study in order to confirm the success of the ischemia procedure and again as a control procedure for the normoxic animals. After the second angiography, the animals were euthanized with potassium chloride infusion under deep anesthesia.

### Device implantation and parameters

Abbott’s Confirm RX DM3500 Insertable Cardiac Monitors (St Jude Medical, Minnesota, USA) were implanted subcutaneously. Confirm RX is a modern ICM device that can detect multiple types of arrhythmias. It has a small size of 9.4 mm × 49 mm, weighing only 3 g. The battery duration is 2 years. Arrhythmia episodes are divided into four main groups: atrial fibrillation (AF), tachycardia, bradycardia, and pause.

**Fig. 1:**
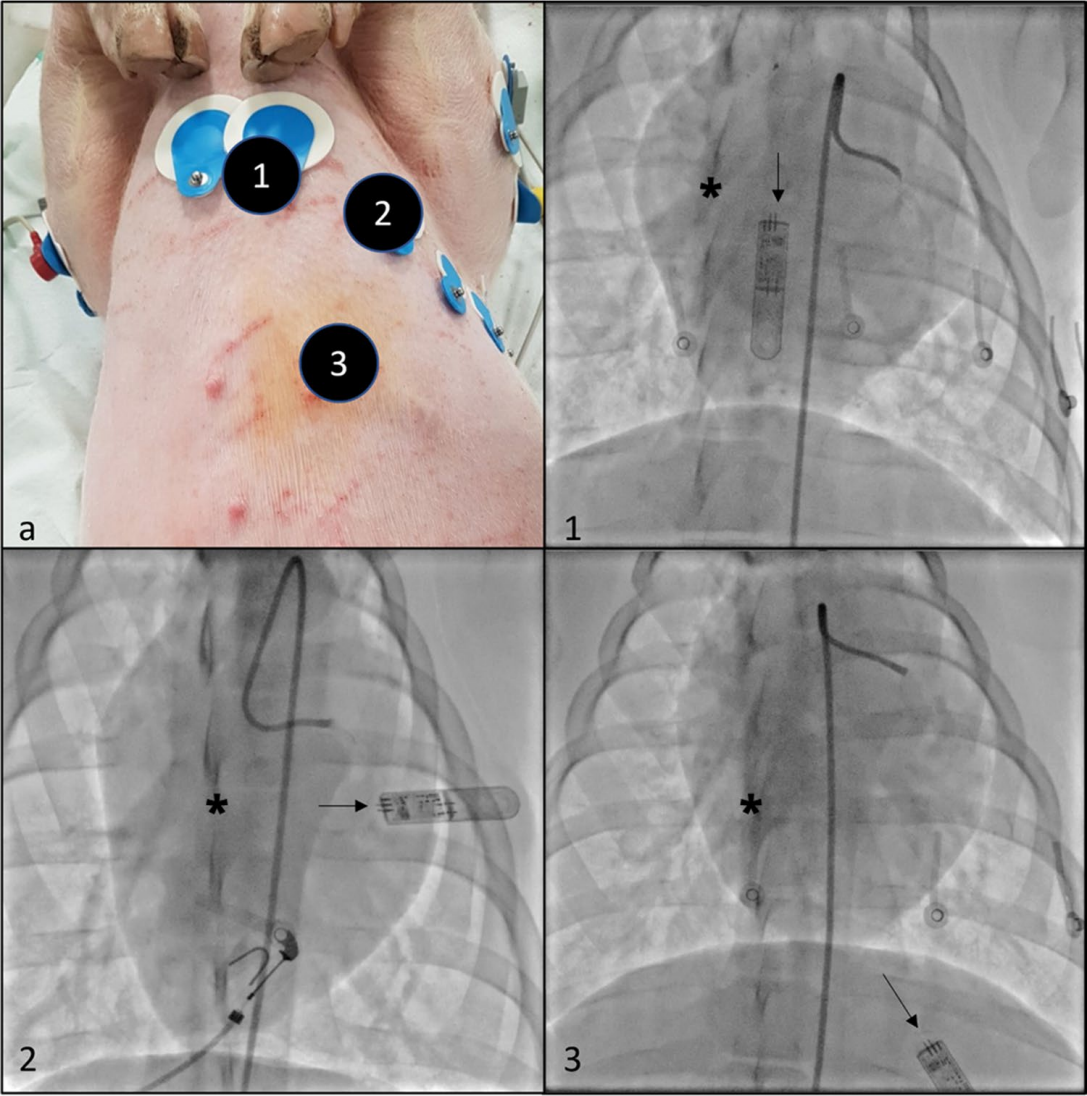
a Implantation and orientation sites. (1) Area on top of the sternum and parasternum. (2) Area on top of the left side ribs. (3) Area immediately below the lowest rib on the left side. 1–3 Angiography images from the anterior–posterior view corresponding to the implantation sites in a. Arrow indicates the ICM position and asterisk indicates the vertebrae. The tip of the angiography catheter is located in the left main coronary artery

Before the ICM implantation, pigs were sedated with an intramuscular injection of 1.5 ml atropine and 6 ml of azaperone. After the sedation, the animals were intubated and propofol anesthesia was induced. The implantation site was mapped with two ECG-electrodes located 4 cm apart, and the highest surface R-wave amplitude was searched. The goal was to test different locations on the thoracic area to estimate the impact of the pig movement on the signal quality. Therefore, multiple implantation sites were tested (Fig. 1).

The implantation site was marked and cleaned. Lidocaine with adrenaline was injected subcutaneously for local anesthetic and carprofen was used as an analgetic. Heart rate, ECG, respiration rate, and oxygen saturation level were monitored throughout the implantation. Cefuroxime 500 mg was given before the operation and on the first postoperative day. Skin puncture was done via the tool provided in the ICM kit. ICM was slid subcutaneously using the manufacturer’s implantation tool. Subcutaneous *R* values were measured and ICM parameters were set accordingly. The minimum amplitude accepted was 0.2 mV. The wound was closed with two sutures and covered with a clean bandage for 2 days.

At first, all the devices had the same programmed parameters. Bradycardia episode threshold was set to ≤ 30 bpm. Pause episode was recorded when no ventricular beats were detected for more than 3 s. AF detection threshold was 1 min, which means that an AF episode was stored if any of the AF detection algorithms reacted at least for a 60-s period. For AF detection, the algorithms estimate for example the sudden changes of R-R intervals, rhythm irregularity, and the non-existence of P-waves. The complexity of AF detection algorithms leads in the storage of multiple types of arrhythmias under the AF category. At first, tachycardia episodes were stored when 12 beats exceeded the threshold of 160 bpm. However, this caused an excessive number of stored episodes. A new threshold was set to 190 bpm with 8–12 consecutive beats required for the episode storage, depending on the R-wave quality. With an R-wave amplitude of higher than 0.4 mV, only 8 beats were required. With an R-wave amplitude of ≤ 0.4 mV 12 beats were needed for the episode storage. R-wave amplitude was also defining the parameters for the dynamic range, sensitivity, and refractory period, and these were adjusted during the follow-up period if significant changes in the amplitude were measured.

### Data collection and analyzing

To protect the wounds, the pigs were separated to individual pens for the first 2 days. However, they could hear, smell, and see their littermates. On the third day, the pigs were moved into herds consisting of 2–3 pigs. ICM memory was interrogated 2–3 times per week in order to avoid overwriting of the episodes. The researcher entered the pen and activated the ICM with a magnet, which connected the device to the external programmer via a Bluetooth connection. Episodes were saved into an external hard drive. The pigs were able to move freely in their pens during the memory reading and the entire follow-up time. Data was collected also during the angiography procedures.

During data collection, some stored episodes were evaluated and R-wave amplitude was measured every time. If needed, ICM parameter changes were made. To detect any signs of infection, the ICM was palpated and the wound area was checked visually daily. Later episodes saved to the external hard drive were manually checked. Both scatter plot and one-lead ECG recording were assessed from the saved episodes to spot true arrhythmias and possible device misinterpretations. From the one-lead ECG, the morphology of P, QRS, and T-waves was evaluated in addition to RR intervals. ICM stores RR interval information to the ECG recording and the accuracy of the device’s interpretation was manually checked. Some measurements, such as the amplitude, were unprecise due to the measurement limitations on the pdf format of the saved episodes.

## Results

The ICMs stored many arrhythmia episodes (Table 1). The total number of episodes per animal varied considerably, mostly due to the different follow-up times, but also due to differences in the signal quality. The episodes contained both correctly recognized arrhythmias and false arrhythmia detections. The false detections were typically triggered either by oversensing or undersensing of the signal. Parameter changes were made during the study if over/undersensing was detected. Typical reason for signal instability was the momentary movement of the device.

**Table 1:** Number of arrhythmia episodes detected during the study. Most episodes were triggered either by oversensing or undersensing of the ECG signal. Pigs 1–6 were normoxic, follow-up 8 days. Pigs 7–13 were ischemic, follow-up 22 days

	Pig 1	Pig 2	Pig 3	Pig 4	Pig 5	Pig 6	Pig 7	Pig 8	Pig 9	Pig 10	Pig 11	Pig 12	Pig 13
AF	34	49	60	5	23	22	43	49	175	115	18	9	89
Tachy	105	26	156	250	56	83	209	53	99	126	168	34	264
Brady	0	0	0	0	0	0	0	0	1	0	0	0	0
Pause	4	3	214	0	0	0	0	40	145	382	2	4	36

### Atrial fibrillation (AF)

**Fig. 2:**
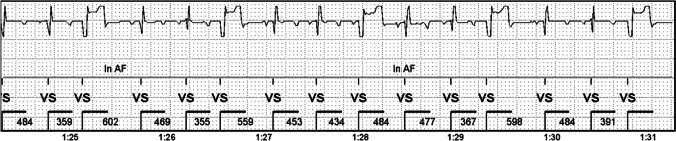
Ventricular trigeminy in a normoxic animal. Trigeminy episode has variation in RR-intervals and results in false AF detection. Negative P-waves can be seen before QRS-complexes

**Fig. 3:**
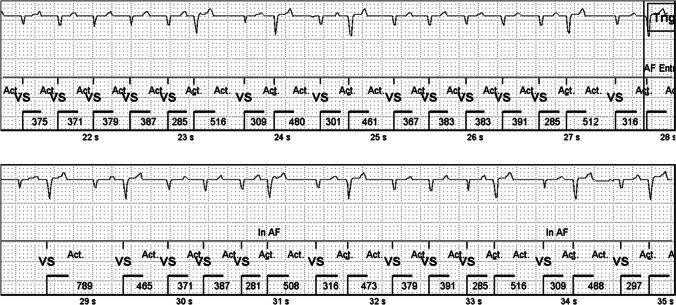
Isolated PVCs in an ischemic animal. PVCs result in irregular rhythm and false AF detection. Notice a good recognition of R-waves despite the quite low QRS amplitude. P-waves are not seen

ICMs classified several arrhythmias into the AF category. If premature ventricular contractions (PVCs) occurred regularly—as in bigeminy or trigeminy—the irregular rhythm frequently resulted in false AF detection (Fig. 2), even though the algorithms should recognize and exclude these patterns. Also, frequent PVCs, though not regular, can be detected as an AF episode (Fig. 3).

The ICMs did not store episodes including only occasional PVCs. In occasional PVCs, the duration of irregular rhythm did not fulfill the threshold of a 60-s AF duration. This was noted during the coronary angiography, when external lead ECG recording was compared with the ICM signal. Sporadic PVCs occurring during angiography did not create an episode in the ICM memory.

**Fig. 4:**
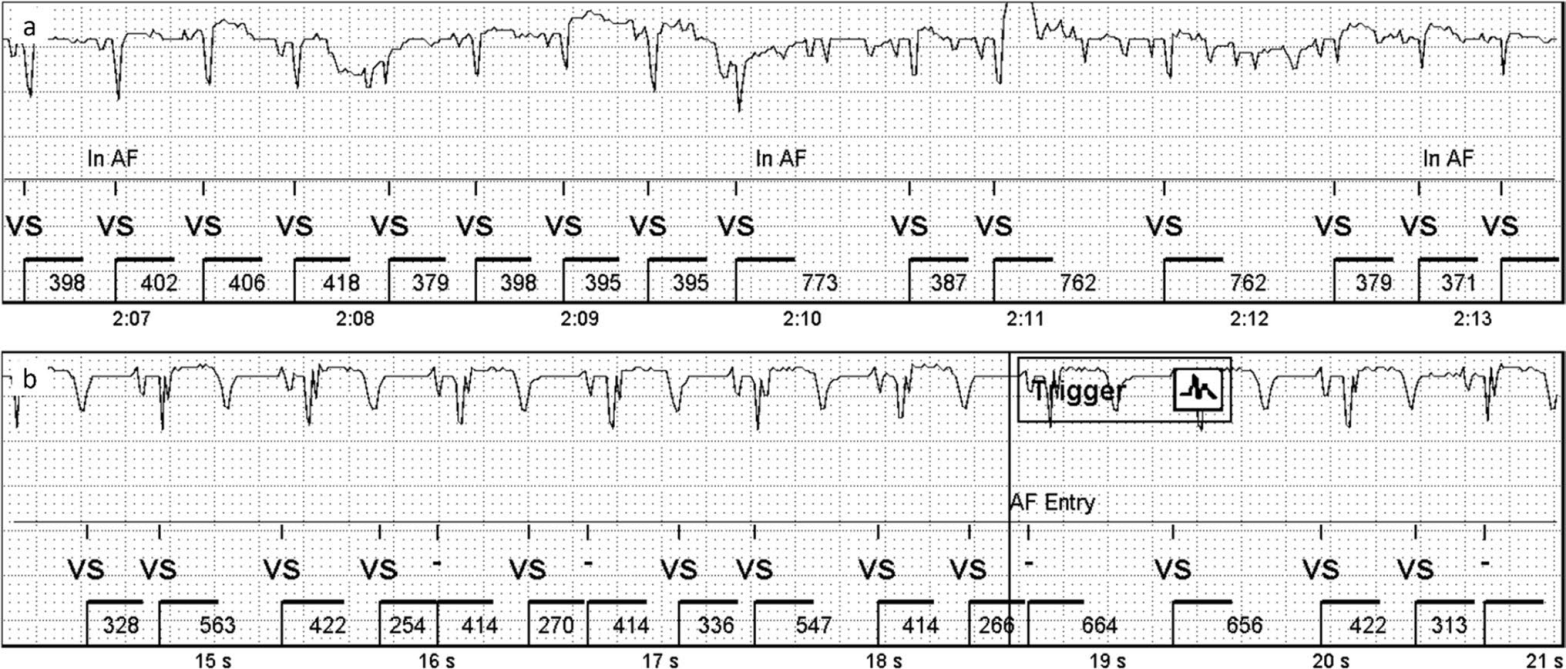
**a** An erratic baseline and amplitude change causing problems in the recognition of P-, R-, and T-waves and leading to false AF detection in an ischemic animal. **b** R-waves are clearly recognizable, but high amplitude P- and T-waves are occasionally detected instead of the R-wave, which disturbs the algorithm. Thus, a regular sinus rhythm is stored as an AF episode in an ischemic animal

Even though ICMs detected multiple arrhythmia types falling into the AF category correctly, most of the stored AF episodes were caused by signal instability and muscle artifacts. In most cases this meant either an erratic baseline deteriorating P-wave recognition and causing undersensing of R-waves due to a low amplitude (Fig. 4a) or occasional oversensing of the T-waves and/or of P-waves (Fig. 4b).

### Tachycardia

**Fig. 5:**
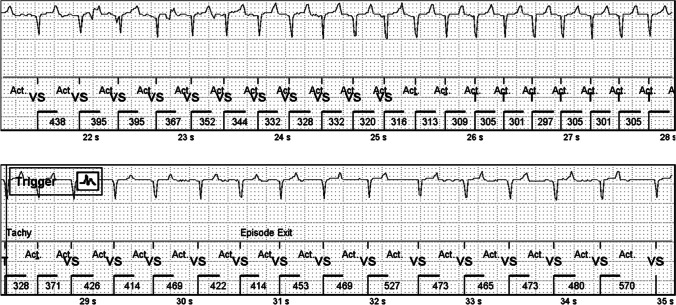
A short tachycardia episode in an ischemic animal. The threshold for tachycardia detection is 190 bpm (316 ms) and 8 intervals. Notice the correctly recognized ventricular depolarization around 22 s despite the disturbances in the ECG signal. All P-waves cannot be seen

Tachycardia episodes were mostly from sinus origin, being normal physiological responses to exercise or stress. At first, tachycardia detection threshold was set to 160 bpm, but this caused an excessive number of sinus tachycardia episode detections. Young pigs have a quite high heart rate around 100 bpm at rest which rises and decreases rapidly, e.g., when moving in the pen. The ICM detects these episodes well (Fig. 5) if the muscle movement does not create too much noise. Threshold of 190 bpm led to a reasonable detection of episodes on most pigs, though still causing many physiological sinus tachycardia registrations. A typical false tachycardia detection was caused by the oversensing of T-waves, which led to doubling of the heart rate.

**Fig. 6:**
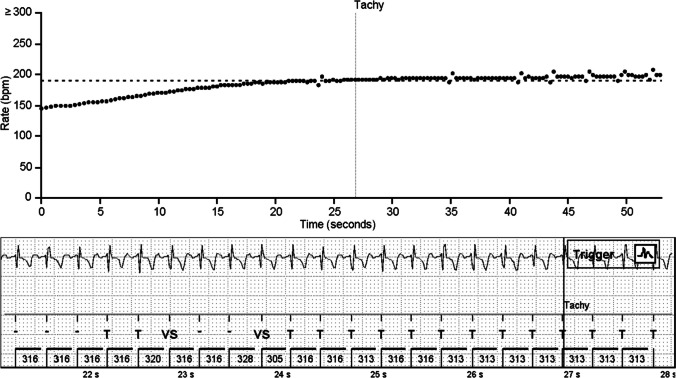
A tachycardia episode in a normoxic animal during dobutamine infusion. The scatter plot shows a continuous increase in the heart rate and exceeding the tachycardia threshold of 190 bpm (dotted horizontal line)

Tachycardia was also pharmacologically provoked by intravenous infusion of dobutamine when the pigs were sedated for angiography. External lead ECG was constantly compared with the ICM signal throughout the operation. ICM could reliably detect these artificially provoked tachycardia episodes and the episodes were saved in the device memory (Fig. 6).

**Fig. 7:**
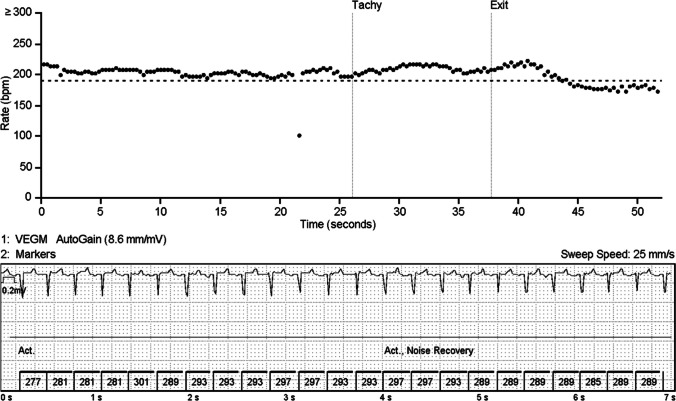
Artifacts caused by muscle noise inhibit the detection of tachycardias in a normoxic animal. The upper figure shows a scatter plot of the tachycardia episode exceeding the threshold of tachycardia detection (dotted horizontal line, 190 bpm) for more than 40 s. However, only a 14-s episode is detected (vertical lines tachy/exit). The reason for this is seen in the lower figure: ICM does not register the VT correctly because of the active noise recovery due to muscle activity. Low amplitude negative P-waves can be seen before some of the QRS complexes. The example is from a normoxic animal

Even though the ICMs usually detected the tachycardias very well, muscle noise impairs the detection of tachycardias (Fig. 7), as well as other types of arrhythmias. The ICM could not detect the rhythm correctly in the presence of muscle noise, and therefore, the ICM algorithms did not spot the episodes that exceeded the pre-set thresholds. When the noise continued throughout the arrhythmia, no episode was stored.

### Bradycardia

Bradycardia detection threshold was 30 bpm for all animals. This created only one bradycardia episode storage during the whole study, and it was due to an underdetection of R-waves.

### Pause

**Fig. 8:**
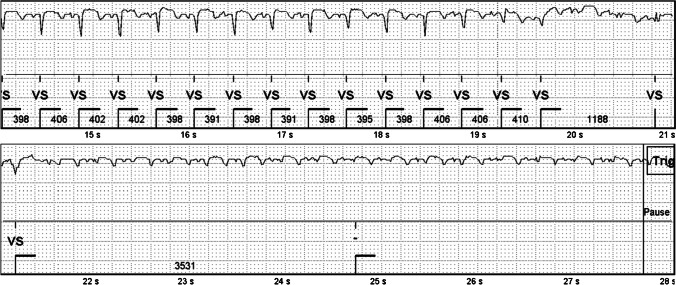
A pause episode in an ischemic animal caused by a poor signal quality, which lasted for 20 s. The root cause was most likely a brief movement of the device

Pause detection threshold was 3.0 s. Typically, a pause episode was caused by a transient decrease in signal quality (Fig. 8) or animal death.

### Implantation site

**Fig. 9:**
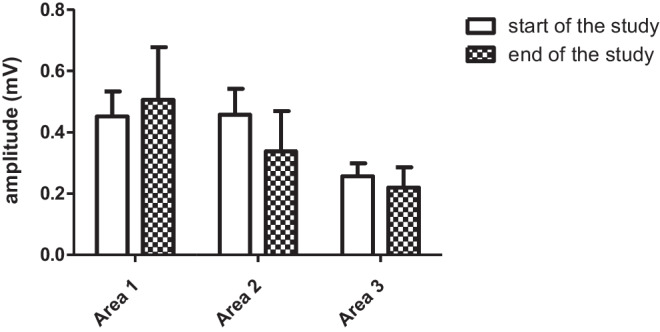
R-wave amplitude in the beginning of the study and at the end of the study in different implantation areas. Area 1 *n* = 5. Area 2 *n* = 5. Area 3 *n* = 3

Choosing the optimal implantation site was a compromise between the signal amplitude and the quality. Figure 1 describes the implantation areas. Figure 9 describes the R-wave amplitudes at the beginning and at the end of the study in the implantation areas.

In area 1, the signal amplitude was good, but not optimal, since it was closer to the right ventricle than the left ventricle. In this set-up, the ICM laid flat on top of the sternum or next to the sternum in a vertical direction. Since pectoralis muscles originate from the sternum, muscle noise could not be completely avoided. Signal strength in this area improved during the study (Fig. 9). This area is commonly used for ICM installations in humans.

The highest signal amplitude was recorded from area 2, but in this area, the muscle noise disturbs the signal quality since it is on top of the pectoralis major and the intercostal muscles. In humans, this area is used for ICM installations even though the muscle noise is present. However, it is notable that pigs move on their four legs, which causes more muscle movement compared to humans. Also, in pigs, the thorax area is more barrel-like compared to humans, which limits the ICM positions. If aiming for an installation position between the ribs, like in humans, the ICM would not lay flat, but both ends would swing unsteadily, which causes the post-implantation amplitude to be slightly lower compared to the surface measurement. Pigs also grow fast, which is a challenge for choosing the ICM position. Signal strength on this area decreased during the study (Fig. 9).

Area 3 had by far the lowest signal amplitude since it was furthest away from the heart. The site was chosen because of the pectoralis muscles not creating noise. However, abdominal muscles originate from this area and muscle noise was still present. Signal strength in this area decreased during the study (Fig. 9). Since this area had the lowest signal amplitude in the beginning of the study, the decrease of the signal during the study caused low amplitude leading to major problems in the detection of arrhythmias.

The longer follow-up group (ischemic pigs) also faced an unexpected problem, when five out of seven ICMs were destroyed by the littermate pigs after 2 weeks. The littermates were constantly poking the ICMs with their snouts, making the devices move subcutaneously, and finally, the devices popped out from the installation wound area. All these five devices were implanted on area 2. New devices were implanted to these pigs to continue the data collection of the ischemic animals.

## Discussion

There are several pre-existing, noninvasive methods available for a short-term ECG and longer Holter recording, and these methods do not cause undesirable animal distress compromising the data [5, 10]. However, these are mostly wearable devices originally designed for human use, which prevent the natural behavior and limit free movement of the animals. With pigs, the animals must be separated to inhibit the littermates from damaging the devices. Also, ECG signal from wearable devices is easily compromised by motion artifacts.

Holter recording is a feasible and most often used electrocardiographic recording for arrhythmia detection, which allows retrospective rhythm evaluation. It is widely used in clinical medicine, and it is applicable to animals as well. The advantages are good availability, automatic recording, and low cost. Holter is a good method to follow heart rhythm for a limited follow-up time (e.g., 48 h) and when screening for frequent arrhythmias. However, a non-anesthetized pig is restless with the Holter device, and the animal itself or the littermates try to remove or destroy the equipment. Therefore, this recording method is not suitable for restless large species such as pigs [9].

Besides Holter, there are more modern techniques for noninvasive ECG recording. External telemetry systems require a telemetry implant and a jacket for continuous data [2]. LifeShirt is a wearable cardiopulmonary monitoring device, which can record continuous ECG and does not restrict animal movement. It is a wearable jacket containing electrodes and transducers that measure ECG, HR, and respiratory activity [11]. However, these jackets limit animals’ behavior and require separate housing to minimize the event of littermates assisting device destruction.

Infrared thermography is a motion-based computer vision method, which can monitor heart rate and respiratory rate, requiring no surgical procedures or implantation of telemetric sensors. HR is estimated by the heart-induced vibrations of the chest and RR by measuring chest movements during respiration. The method is easy and it does not need light in the room, but on the downside, it is expensive and not accurate for the detection of arrhythmias in the pigs [1].

Invasive intracardiac ECG data collection methods also exist, but they require anesthesia and a recovery period. Accelerometers are motion sensors which can be used for postoperative monitoring after cardiac surgery, as they provide information on the heart wall motion and cardiac function. They need implantation of a pacemaker electrode in the heart [4].

Modern pacemakers are able to record periodic ECG data [3]. Pacemakers are accurate in evaluating arrhythmias [7] but the implantation is highly invasive. The procedure itself requires more time and expertise than the ICM, and in addition, the implantation is done under fluoroscopy guidance. Pacemaker electrodes are implanted in the heart and the generator in the chest subcutaneously. The incision through which the generator is placed is easily exposed by littermates and gets easily infected.

Compared to these other ECG monitoring methods, ICMs are minimally invasive, suitable for long-term monitoring, and allow reliable monitoring in pigs. In general, ICMs record and recognize different arrhythmias from pigs very well. The ICM implantation is also quick and easy, and the risk of local pocket infection is low.

However, the ICMs tend to overanalyze T-waves, leading to oversensing. Sometimes the R-waves might be left unnoticed due to the ICM movement, leading to the false registration of AF or pause. Also, high heart rates in the pigs might lead to false detection of tachycardias. In spite of these shortcomings, the false registrations are usually easy to recognize when analyzing the episodes. The major downside is that noise recording can fill the memory of the device. However, atrial rhythm is often difficult to assess because there is only a single-lead ECG available, and this might complicate the recognition of the arrhythmia. Also, the ICMs are not reliable in identifying single extrasystoles, unless the extra beats occur very densely.

One obvious challenge is the heterogeneous signal. Muscle contractions cause noise, interfering with real arrhythmias. This downside is widely recognized in human use, too. Pigs naturally get easily excited or startled, affecting the ECG. The pigs tend to scratch the implantation sites, causing the ICM to move or to erose through the skin. The position change affects the quality of the signal and leads to the need of adjusting the parameters. A deeper implantation site under the adipose tissue might give a better signal.

## Conclusions

In conclusion, ICMs can detect most arrhythmia types in pigs and are suitable for long-term rhythm monitoring. Both oversensing and undersensing occur, but these can be avoided with parameter optimization and manual evaluation when analyzing the episodes. The device implantation is fast and simple, and the subcutaneous location does not limit the movement of the animals. Other minimally invasive methods are usable only for a shorter period, they often limit the animal behavior and face similar ECG signal disturbances as ICMs. Other more accurate methods are highly invasive. Thus, ICMs are useful devices for minimally invasive long-term monitoring of cardiac rhythm in pigs.

## Data Availability

The datasets analyzed during the study are available from the corresponding author on reasonable request.

## Abbreviations

*ICM*: Insertable cardiac monitor
*AF*: Atrial fibrillation
*PVC*: Premature ventricular contraction
*VT*: Ventricular tachycardia
*ECG*: Electrocardiogram
